# Diabetes and osteoporosis: a two-sample mendelian randomization study

**DOI:** 10.1186/s12891-024-07430-0

**Published:** 2024-04-23

**Authors:** Yu-Dun Qu, Zhao-Hua Zhu, Jia-Xuan Li, Wei Zhang, Qi Chen, Chang-Liang Xia, Jun-Nan Ma, Shuan-Ji Ou, Yang Yang, Yong Qi, Chang-Peng Xu

**Affiliations:** 1 The Second School of Clinical Medicine, Guangdong Second Provincial General Hospital, Southern Medical University, Guangzhou, Guangdong P.R. China; 2 Clinical Research Centre, Zhujiang Hospital, Southern Medical University, Guangzhou, Guangdong China; 3 Department of Orthopaedics, Guangdong Second Provincial General Hospital, The Second School of Clinical Medicine, Southern Medical University, Guangzhou, Guangdong P.R. China; 4 Department of Orthopaedics, Guangdong Second Provincial General Hospital, No. 466 Xingang Road, Haizhu District, Guangzhou, 510317 Guangdong People’s Republic of China

**Keywords:** Diabetes, Bone mineral density, Osteoporosis, Fracture, Mendelian randomization

## Abstract

**Background:**

The effects on bone mineral density (BMD)/fracture between type 1 (T1D) and type 2 (T2D) diabetes are unknown. Therefore, we aimed to investigate the causal relationship between the two types of diabetes and BMD/fracture using a Mendelian randomization (MR) design.

**Methods:**

A two-sample MR study was conducted to examine the causal relationship between diabetes and BMD/fracture, with three phenotypes (T1D, T2D, and glycosylated hemoglobin [HbA1c]) of diabetes as exposures and five phenotypes (femoral neck BMD [FN-BMD], lumbar spine BMD [LS-BMD], heel-BMD, total body BMD [TB-BMD], and fracture) as outcomes, combining MR-Egger, weighted median, simple mode, and inverse variance weighted (IVW) sensitivity assessments. Additionally, horizontal pleiotropy was evaluated and corrected using the residual sum and outlier approaches.

**Results:**

The IVW method showed that genetically predicted T1D was negatively associated with TB-BMD (*β* = -0.018, 95% CI: -0.030, -0.006), while T2D was positively associated with FN-BMD (*β* = 0.033, 95% CI: 0.003, 0.062), heel-BMD (*β* = 0.018, 95% CI: 0.006, 0.031), and TB-BMD (*β* = 0.050, 95% CI: 0.022, 0.079). Further, HbA1c was not associated with the five outcomes (*β* ranged from − 0.012 to 0.075).

**Conclusions:**

Our results showed that T1D and T2D have different effects on BMD at the genetic level. BMD decreased in patients with T1D and increased in those with T2D. These findings highlight the complex interplay between diabetes and bone health, suggesting potential age-specific effects and genetic influences. To better understand the mechanisms of bone metabolism in patients with diabetes, further longitudinal studies are required to explain BMD changes in different types of diabetes.

**Supplementary Information:**

The online version contains supplementary material available at 10.1186/s12891-024-07430-0.

## Background

Diabetes mellitus, a prevalent noncommunicable chronic disease [International Diabetes Federation (IDF), Available at: http://www.diabetesatlas.org], poses a global public health challenge and is associated with severe disability and mortality [1]. In 2019, its worldwide prevalence was approximately 9.3%, rising to 9.6% in the Western Pacific, and is projected to affect 693 million individuals by 2045, a 50% increase from 2017 [2]. The two main forms of diabetes are type 1 (T1D) and type 2 (T2D). T1D is caused by the immune system’s elimination of pancreatic beta cells [3], which results in a dramatic decrease in blood insulin levels. Contrarily, T2D is more prevalent among adults and older adults and constitutes roughly 90% of all cases of diabetes [4]. Morbidity and mortality in diabetes mainly stem from complications of the macrovascular (cardiovascular disease) and microvascular (diabetic kidney disease), retinopathy, and neuropathy systems [5].

Osteoporosis (OP) is characterized by low bone mass and altered bone architecture, leading to compromised bone strength and an increased risk of fracture [6]. Often termed a “silent disease,” OP frequently manifests no signs until the occurrence of the first fracture [7]. Clinically, OP is diagnosed by measuring bone mineral density (BMD), with a T score of -2.5 as the cutoff [8], which remains the strongest predictor of fracture risk. Globally, an osteoporotic fracture occurs every 3 s, resulting in over 8.9 million fractures annually [9]. The disease inflicts considerable emotional, physical, and financial burden on patients, often leading to disability, diminished quality of life, and mortality [10]. Furthermore, the prevalence of both diabetes and OP is on the rise due to population aging and increased life expectancy among patients with diabetes [11].

Over 60 years ago, Albright and Reifenstein proposed a potential link between diabetes mellitus and OP, suggesting that diabetes might contribute to bone mass loss leading to OP [12]. This topic has since garnered significant attention and investigation [13]. A 2019 observational study involving 9238 adults with diabetes and 99,980 individuals without diabetes found a significant association between diabetes and OP (1.2 [1.1–1.4], *P* = 0.010) [14]. Specifically, diabetes was linked to a decreased BMD, elevating the risk of bone fracture [15]. While most studies suggest a modest reduction in BMD associated with diabetes [16], some do not confirm this finding [17]. Recent cohort studies have indicated a 4–12-fold increase in the risk of hip fracture among individuals with diabetes [18, 19], contrasting earlier case-control investigations that found no elevated risk [20, 21].

To evaluate potential causal links, Mendelian randomization (MR) offers a valuable alternative method [22]. It aims to reduce confounding effects and prevent reverse causation bias since genotypes are independent of postnatal lifestyle and environmental variables and precede the onset of disease [23]. To investigate the quantitative impact of diabetes (both T1D and T2D) and associated glycemic characteristics (glycosylated hemoglobin [HbA1c]) on various aspects of bone health, including femoral neck BMD (FN-BMD), lumbar spine BMD (LS-BMD), total body BMD (TB-BMD), and fracture, we conducted an analysis using MR and genome-wide association study (GWAS) data analysis.

## Methods

### Study design and data sources

**Fig. 1:**
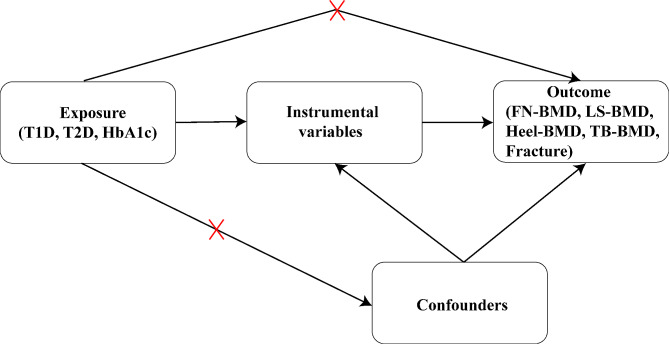
Illustration of the two-sample Mendelian randomization analysis. T1D: type 1 diabetes; T2D: type 2 diabetes; HbA1c: glycosylated hemoglobin; FN: femoral neck; LS: lumbar spine; TB: total body

**Fig. 2:**
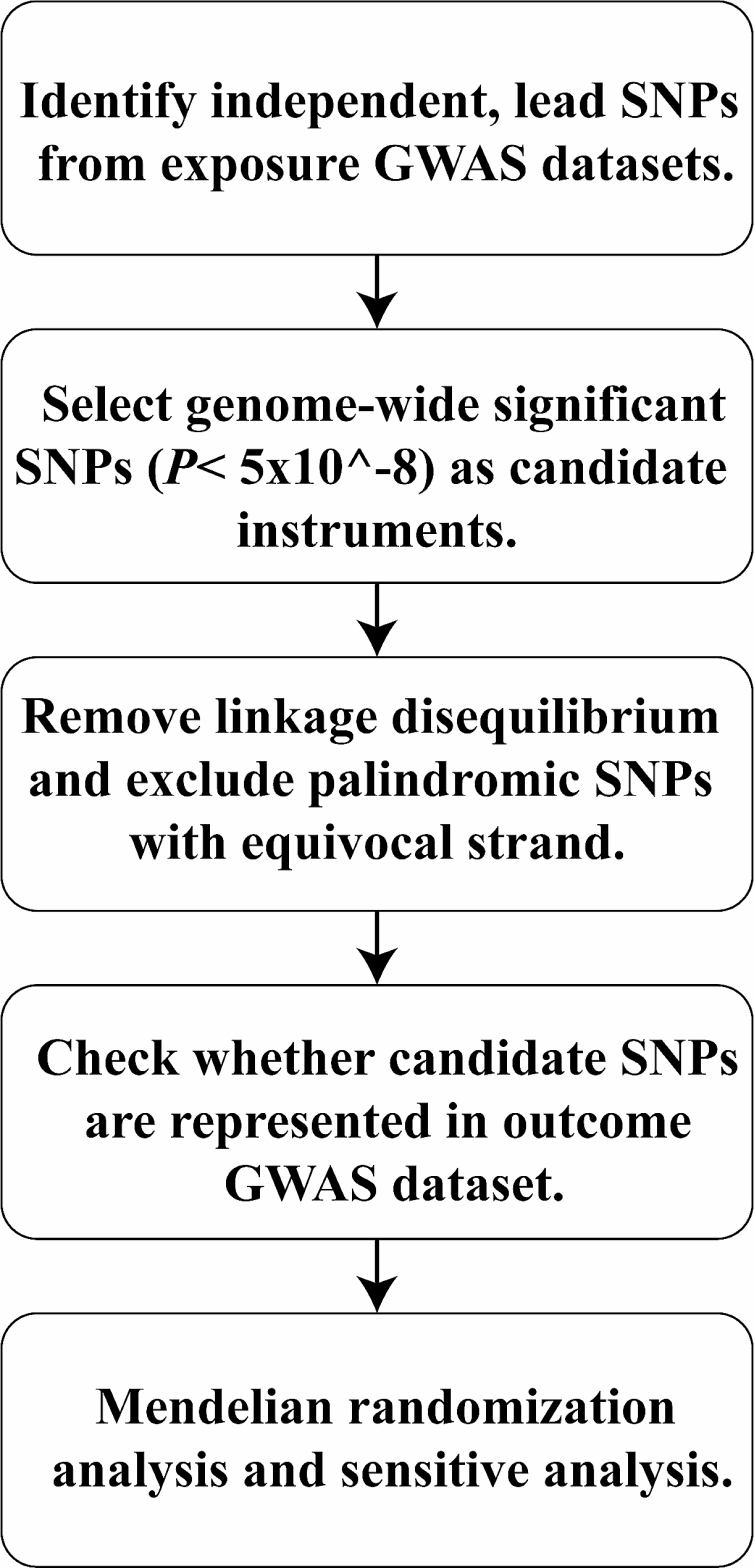
Flowchart of a Mendelian randomization research. GWAS: Genome wide association study; SNPs: Single-nucleotide polymorphisms

In 2021, the American Diabetes Association released the following new diagnostic criteria for diabetes [24]: (1) fasting plasma glucose level ≥ 126 mg/dL (7.0 mmol/L), (2) 2-h plasma glucose level ≥ 200 mg/dL (11.1 mmol/L) during Diabetes Control and Complications Trial (OGTT), (3) HbA1c level ≥ 6.5% (48 mmol/mol), and (4) random plasma glucose level ≥ 200 mg/dL (11.1 mmol/L). Compared to T2D, T1D typically presents at a younger age at diagnosis (< 35 years) with a lower body mass index (BMI, < 25 kg/m^2^) and is characterized by positivity for insulin-related antibodies [25]. This study employs a two-sample MR design adhering to STROBE-MR guidelines [26] (Figs. 1 and 2). Utilizing publicly available summary statistics from GWAS consortia, MR leverages genetic variants associated with the exposures of interest to examine their associations with disease outcomes. As genetic predisposition to a trait is not affected by potential confounders, this approach is considered to be less prone to confounding compared to traditional observational analyses.

### Selection of instrumental variables

Using this strategy, we examined GWAS data from the largest investigations of the genetic causes of T1D that are currently available (*N* = 15,573 cases and *N* = 158,408 controls) [27], T2D (*N* = 265,678 cases) [28], and HbA1c (*N* = 46,368 cases) [29]. Three types of genetic susceptibility to diabetes were adjusted for age, sex, weight, and height. Five phenotypes (FN-BMD, LS-BMD, heel-BMD, TB-BMD, and fracture) were used as outcomes. Table 1 presents additional details of the exposures and outcomes.

**Table 1:** Summary of two-sample Mendelian randomization analyses

Exposure		Outcome	
Diabetes	Source	BMD/fracture	Source
**T1D**	Summary-level for T1D [27], *N* = 5913 cases diagnosed before the age of 17 years and *N* = 8828 controls	**FN-BMD and LS-BMD**	Summary-level FN-BMD and LS-BMD [30], FN (*N* = 32,735), LS (*N* = 28,498)
		**TB-BMD**	Summary-level for TB-BMD [31], (*N* = 66,628)
**T2D**	Summary-level for T2D [28], *N* = 265,678 cases	**Heel-BMD and Fracture**	Summary-level heel-BMD [32], (*N* = 426,824) Summary-level fracture [32], (*N* = 416,795)
**HbA1c**	Summary-level for HbA1c [29], *N* = 46,368 cases		

T1D: type 1 diabetes; T2D: type 2 diabetes; HbA1c: glycosylated hemoglobin; BMD: bone mineral density; FN: femoral neck; LS: lumbar spine; TB: total body; HbA1c: glycosylated hemoglobin.

For MR estimates, instrumental variables (IVs) were derived from summary-level GWAS data. Genetic variations linked to diabetes were identified as instrumental single nucleotide polymorphisms (SNPs). From our analyses (Table 1), we selected a set of independent genome-wide significant genetic variants for T1D, T2D, and HbA1c as IVs (*P* < 5 × 10^− 8^). We used linkage disequilibrium (LD) [33] clustering with a threshold of r^2^ > 0.001 and excluded variations within a 1 Mb distance from other SNPs with stronger connections to ensure independence among instrumental SNPs for each exposure. Additionally, we standardized the effects of these instrumental SNPs whenever feasible and excluded those not present in the GWAS of the outcomes to ensure alignment of all associated risk factors and resulting alleles on the same strand. Ultimately, we selected SNPs associated with each exposure (T1D = 47, T2D = 57 and HbA1c = 10).

### Associations with outcomes

Clinically, OP is defined by measuring BMD with a T score below − 2.5, which remains the single best predictor of fractures [34]. In this research, BMD and fracture data were used to characterize OP phenotypes at various anatomical sites, including FN (*n* = 32,735), LS (*n* = 28,498), systemic TB (*n* = 66,628), heel (*n* = 426,824), and fracture (*n* = 416,795). Data from the Genetic Factors for Osteoporosis meta-analysis of FN-BMD and LS-BMD in the European population (32,735 and 28,498 individuals, respectively, in 2012) were included [35]. Additionally, summary statistics from a GWAS meta-analysis involving 66,628 European participants were included for TB-BMD [31]. The UK Biobank conducted a comprehensive study on the genetic influences on human heel-BMD and fracture risk, encompassing 426,824 and 416,795 individuals for the GWAS dataset for heel-BMD and fracture [32], respectively. Further, BMD phenotypes (per standard deviation) were adjusted for age, sex, weight, and height in the previous GWAS studies.

### MR analysis

The causal relationship between each exposure and outcome was assessed using the inverse variance weighted (IVW) approach with a fixed-effect model. We excluded IVs that were substantially linked with outcome. The IVW approach is often considered the most reliable indicator in MR analysis when evidence of directional pleiotropy is absent (*P* for MR-Egger intercept > 0.05). When each genetic variation meets the IV hypothesis, the IVW method can yield a consistent estimate of the exposure’s causal effect on the outcome. Cochran’s Q statistics were employed to evaluate the IV heterogeneity.

To further validate MR estimates, we used the MR-Pleiotropy Residual Sum and Outlier (MR-PRESSO) techniques, which identify and remove potential pleiotropic IVs, providing outlier-adjusted estimates for IVW analysis (*P* < 0.05). Additionally, we employed complementary analysis methods including weighted median, MR-Egger, simple mode, and weighted mode, using random-effect model estimation to test the robustness of the IVW method. The weighted median estimate, utilizing aggregate data, offers protection against ineffective instruments and provides reliable estimates of causation if at least 50% of the weight originates from IVs [36].

### Sensitivity analysis

In addition to employing the MR-PRESSO technique to identify and eliminate potential pleiotropic IVs and offer outlier-adjusted estimates to the IVs for IVW analysis (*P* < 0.05), we used the Single Nucleotide Polymorphisms Annotator tool (https://snipa.helmholtz-muenchen.de/snipa3/) to analyze the pleiotropy of potential confounders [37]. Cochran’s Q statistics were utilized to evaluate the IV heterogeneity, and loci displaying considerable heterogeneity were eliminated to further confirm the reliability of our MR estimations. Furthermore, we employed weighted median, weighted mode, simple mode, and MR-Egger as complementary analysis methods to assess the robustness of the IVW method using random-effect model estimation.

### Subgroup analysis of age

Considering the potential impact of using summary data from GWAS analyses across all age groups on the accuracy of MR analyses, we performed a more detailed analysis by age groups for TB-BMD. Further, TB-BMD data were stratified into five age stages: 0–15 years (*N* = 11,807), 15–30 years (*N* = 4180), 30–45 years (*N* = 10,062), 45–60 years (*N* = 18,805), and over 60 years (*N* = 22,504). The MR analysis method was employed to estimate the correlation between diabetes and TB-BMD at each respective age stage.

## Results

### MR analysis of the effects of diabetes on BMD/fracture

In our initial investigation, we assessed the causal associations between diabetes and BMD/fracture utilizing a two-sample MR approach. We identified 47 IVs for T1D, 57 for T2D, and 10 for HbA1c, all of which reached genome-wide significance (*P* < 5 × 10^− 8^) in GWAS analyses after removing some missing data. These IVs were selected based on their independence from LD effects (r^2^ < 0.001). Heterogeneity tests revealed no significant heterogeneity among the selected IVs (Q-*P* > 0.05, Supplementary Tables S1-S3 in Additional file 1), demonstrating that neither horizontal pleiotropy nor heterogeneity influenced our MR results. Additionally, supplementary Tables S6-S8 in Additional file 1 provide details on the power of selected IVs.

**Fig. 3:**
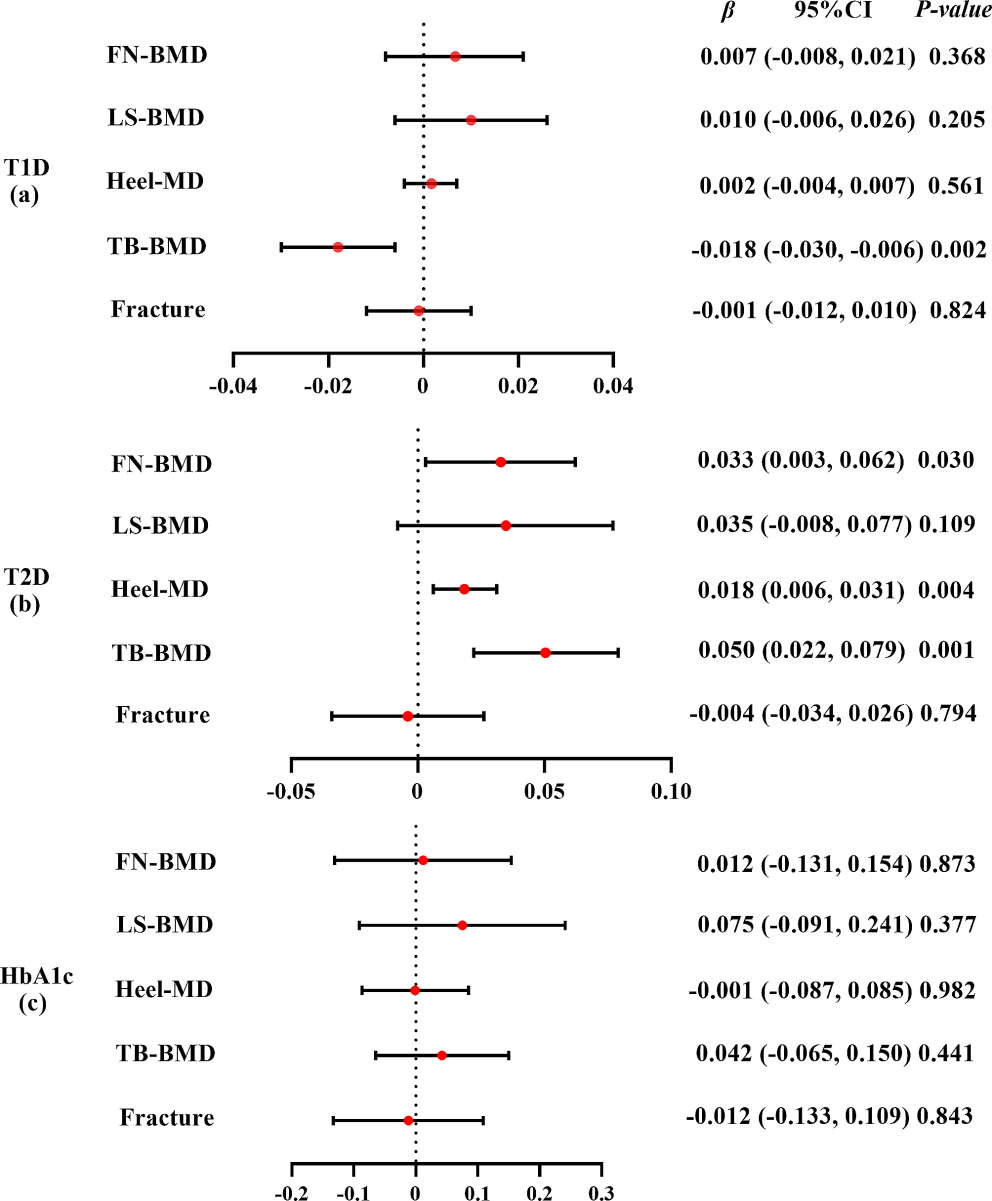
Mendelian randomization estimates the effect of T1D/T2D/HbA1c risk on bone mineral density and fracture. T1D: type 1 diabetes; T2D: type 2 diabetes; HbA1c: glycosylated hemoglobin; FN: femoral neck; LS: lumbar spine; TB: total body; BMD: bone mineral density. Detailed data are provided in Additional file 1 (Supplementary Tables S1–S3)

The IVW method showed that genetically predicted T1D was negatively associated with TB-BMD (*β* = -0.018, 95% CI: -0.030, -0.006) (Fig. 3(a)). Consistent findings were observed with causal estimates from MR-Egger, weighted median, and weighted mode for T1D’s effect on TB-BMD. Although T1D displayed heterogeneity for TB-BMD (Cochran’s Q-*P* < 0.05), the heterogeneity was reduced after processing using the random effect model (IVW, *P* > 0.05). The MR-PRESSO test showed no horizontal pleiotropic effect (*P* > 0.05). Interestingly, genetically predicted T1D was not associated with FN-BMD (*β* = 0.007, 95% CI: -0.008, 0.021), LS-BMD (*β* = 0.010, 95% CI: -0.006, 0.026), heel-BMD (*β* = 0.002, 95% CI: -0.004, 0.007), or fracture (*β* = -0.001, 95% CI: -0.012, 0.010) (Supplementary Table S1 in Additional file 1).

The IVW method showed that genetically predicted T2D was positively associated with FN-BMD (*β* = 0.033, 95% CI: 0.003, 0.062), heel-BMD (*β* = 0.018, 95% CI: 0.006, 0.031), and TB-BMD (*β* = 0.050, 95% CI: 0.022, 0.079) but not with LS-BMD (*β* = 0.035, 95% CI: -0.008, 0.077) or fracture (*β* = -0.004, 95% CI: -0.034, 0.026) (Fig. 3(b)). The causative effect of T2D on heel-BMD was disclosed by causal estimates from MR-Egger and weighted median, and the causal effect of T2D on TB-BMD was also indicated by causal estimates from weighted median and weighted mode. The heterogeneity (Cochran’s Q-*P* < 0.05) of T2D on heel-BMD and TB-BMD existed and was persistent after processing using the random effect model (IVW *P* < 0.05). The MR-PRESSO test showed no horizontal pleiotropic effect (*P* > 0.05). Weighted mode showed potential causal association between T2D and FN-BMD (*β* = 0.053, 95% CI: 0.005, 0.100). However, no causal relationship between T2D and FN-BMD, LS-BMD, TB-BMD, or fracture was observed by MR-Egger (Supplementary Table S2 in Additional file 1).

The IVW method showed that genetically predicted HbA1c was not associated with the five outcomes (*β* ranged from − 0.012 to 0.075) (Fig. 3(c)). The causal estimates from MR-Egger, weighted median, simple mode, and weighted mode were highly similar (*β* ranged from − 0.183 to 0.075) (Supplementary Table S3 in Additional file 1). Although HbA1c displayed heterogeneity for heel-BMD (Cochran’s Q-*P* < 0.05), the heterogeneity reduced after processing using the random effect model (IVW, *P* > 0.05). The MR-PRESSO test showed no horizontal pleiotropic effect (*P* > 0.05).

### Subgroup analysis by age

**Fig. 4:**
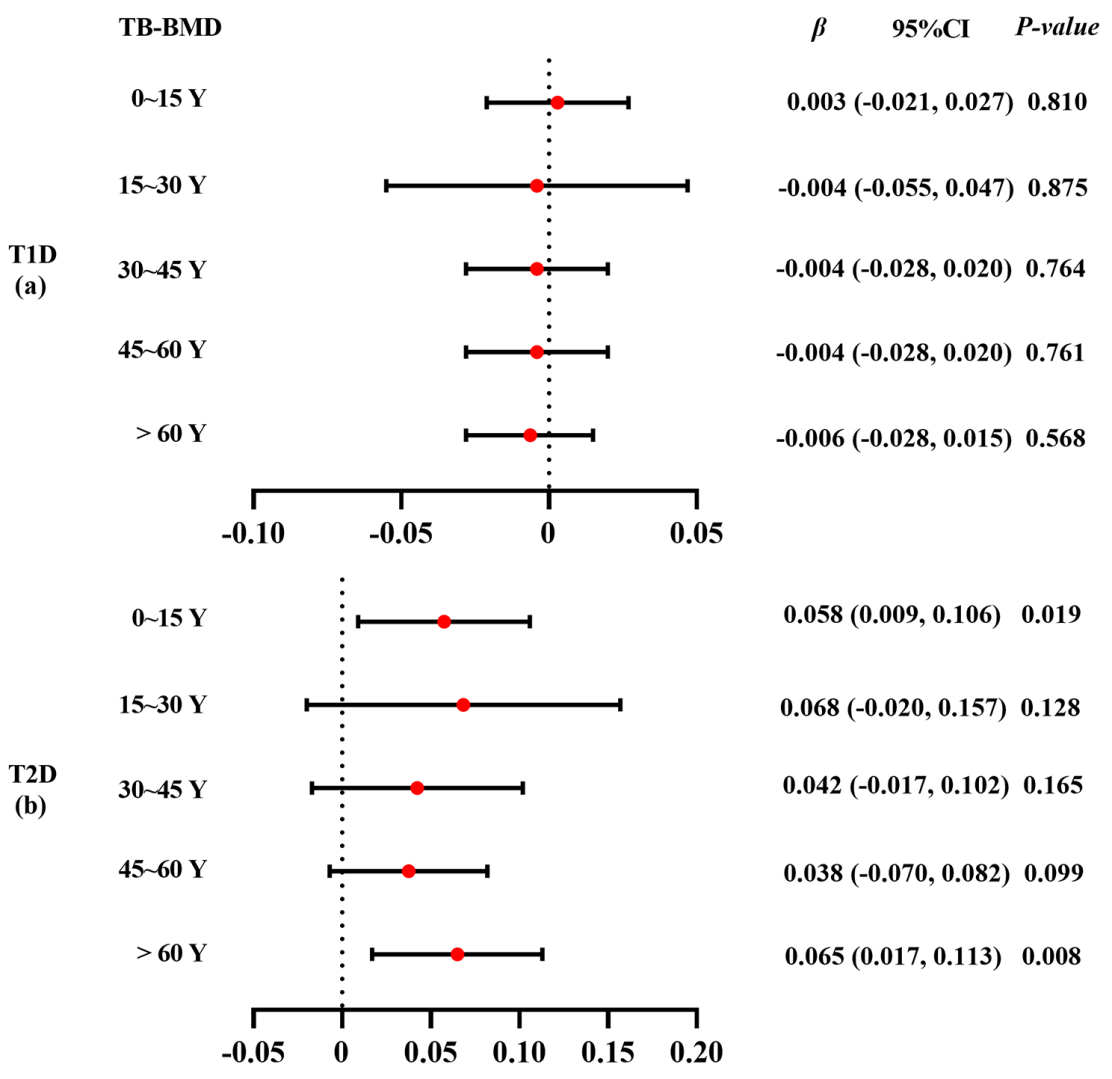
Subgroup analysis using Mendelian randomization estimates for T1D/T2D on total-body bone mineral density by age. TB: total body; BMD: bone mineral density. Detailed data are provided in Additional file 1 (Supplementary Tables S4–S5)

Results of the subgroup analyses of the association between T1D and TB-BMD according to age are shown in Fig. 4 (a). The weighted median indicated a causal association between T1D and TB-BMD (*β* = -0.027, 95% CI: -0.052, -0.002) between 45 and 60 years of age. However, causal estimates from MR-Egger, weighted median, simple mode, and weighted mode did not reveal a significant association between them. The estimates from IVW method, MR-Egger, simple median, and weighted median showed that genetically predicted T1D was not associated with any of the five outcomes (*β* ranged from − 0.006 to 0.181) (Supplementary Table S4 in Additional file 1).

Results of the subgroup analyses of the association between T2D and TB-BMD according to age are presented in Fig. 4 (b). The IVW method showed that genetically predicted T2D was positively associated with TB-BMD under 15 years (*β* = 0.058, 95% CI: 0.009, 0.106). The causal estimates from IVW, weighted median, and weighted mode revealed a causal association between T2D and TB-BMD in individuals over 60 years. However, no causal effect of T2D and TB-BMD was found in individuals aged 15–60-years (Supplementary Table S5 in Additional file 1).

## Discussion

Using a two-sample MR method, we revealed a causal association of T1D and T2D risk with TB-BMD. Additionally, we found that T2D risk was associated with FN-BMD and heel-BMD. However, no clear evidence of a causal relationship between HbA1c and OP/fracture was found. The subgroup analyses by age revealed that T2D has a stronger causal effect on TB-BMD than does T1D, especially in individuals aged > 60 years. Notably, this MR study yielded consistent results even after adjusting for diabetes-related characteristics.

Our study indicated that genetically increased T1D risk was significantly associated with decreased TB-BMD but not with other outcomes (FN-BMD, LS-BMD, and heel-BMD)/fracture. Although T1D commonly occurs between the ages of 9 and 14 years [38], a critical period for optimal bone development in children and adolescents [39], subgroup analysis by age revealed that the causal effect of T1D on TB-BMD was only evident in individuals aged 45–60 years.

A case-control study involving 32 children with T1D found that TB-BMD was significantly reduced in children with T1D, but not in the lumbar spine, suggesting a negative association between T1D and TB-BMD, albeit with different ages [40]. Similarly, a 2021 cross-sectional study of BMD using dual X-ray densitometry at the femoral neck, entire hip, lumbar spine, and whole body reported that men with T1D had compromised bone material strength and microarchitecture, and individuals with T1D showed a modest decline in BMD and low bone turnover [41]. Other studies have also found reduced BMD in patients with T1D [42]. However, a newly published MR study did not find an association between T1D and fracture/OP [43]. Although previous studies have revealed that the deficiency in bone mass in T1D may be apparent at the time of diagnosis [44], whether the duration of the disease or any other clinical aspects of diabetes or its management are linked to unfavorable skeletal morbidity remains unknown. Increasingly, experts recognize that a thorough evaluation of bone health necessitates examining bone microarchitecture alongside bone density. This recognition stems from the fact that the greatly increased fracture risk in individuals with T1D is disproportionate to the barely detectable decline in BMD [45].

While T2D demonstrated no significant association with LS-BMD or fracture risk, a notable association was observed between genetically predicted T2D and FN-BMD, heel-BMD, and TB-BMD. Recently, an MR study also found that T2D can reduce the incidence of OP, that is, increase BMD (*P* = 0.0056) [46]. The 2017 MR study on T2D and BMD revealed a weak positive correlation between the two, whereas no link between T2D and LS-BMD was reported, and these findings were independent to BMI [47]. Secondary analysis of a cross-sectional data from youth aged 10–23 years (55% African American, 70% female) with T2D (*N* = 90), obesity (BMI > 95th; *N* = 128), or normal weight (BMI 85th; *N* = 197) revealed that the obesity and T2D groups had significantly higher BMD Z-scores than those of the normal weight group [48]. Given that T2D may have an asymptomatic phase prior to diagnosis, we observed an increase in BMD around the time of T2D diagnosis [49]. However, we did not find evidence of increased heel-BMD in the T2D population, suggesting the need for further investigation to confirm this observation. Notably, subgroup analysis by age showed that the causal effect of T2D on TB-BMD was stronger in individuals over 60 years than in those under 15 years. This finding is supported by a prospective observational study [50] in which all patients with T2D newly diagnosed through normal health care procedures were identified between May 1, 1996 and June 30, 1998. The mean age at T2D diagnosis was 62.9 years for men and higher for women [50]. The considerable regional variation in bone microstructure across the skeleton and the known distinct effects of T2D on the cortical and trabecular bones [42] may be associated with a suggested mechanism for site-specific effects of T2D on BMD [51]. Several hypotheses exist regarding the correlation between T2D and BMD and bone quality. One hypothesis suggests that low bone mass results from insulin insufficiency in type-1 diabetes, but increasing insulin levels in individuals with T2D may increase BMD because insulin signals the bone’s osteoblasts to become more active [52]. However, patients with T2D may develop insulin resistance, which may compromise the physiological effects of insulin on bones. Additionally, it has been hypothesized that hyperglycemia, which characterizes T2D [53], impacts bone integrity, presumably by elevating the quantities of advanced glycation end products and collagen cross-linking, which have been associated with an increased risk of fractures [54]. . A 2019 prospective cohort study offers a relatively plausible explanation for the Diabetes Bone Paradox [55] (high risk of fracture but normal or increased BMD) in type 2 diabetes [56]. In a previous study, frailty status was measured by the Camos-based algorithm for Frailty Index (FI) of deficit accumulation [57]. Guowei Li et al. found that the increased fracture risk in patients with T2D may be related to the frailty of the patients themselves, and T2D exacerbates this process.

In the present MR analysis, HbA1c did not show a causal effect on FN-BMD, LS-BMD, heel-BMD, TB-BMD, and fracture. This observation implies that the influence of metabolic management on BMD may not be directly proportional over extended durations. This is because the detrimental effects of diabetes on BMD may have a more substantial impact than what can be solely attributed to levels of HbA1C [58]. Although long-term glycemic management is not linked to BMD, this does not rule out the possibility that patients with T1D may experience short-term, reversible alterations in BMD that can be treated to reverse them [58].

Notably, this study revealed that T1D had a weak negative correlation with BMD, whereas T2D had a positive correlation with BMD. Recent research, including two meta-analyses, has demonstrated a considerably higher risk of fracture (six studies, 35,925 individuals with T1D), reduced BMD (16 studies, 966 adults with T2D), and OP in individuals with T1D [59]. The status of BMD in patients with T2D is debatable, and some meta-analyses have found that the risk of fractures is high in patients with T2D [60]. The risk of hip fracture has been shown to increase by 1.3–2.1 times [61], and the risk of other fractures reportedly increases by 1.2 times [60], while the risk of vertebral fractures does not increase [60]. Further, BMD is enhanced in patients with T2D (lumbar spine Z-score + 0.41, total hip Z-score + 0.27), even though the fracture risk is high [62]. Considering that nearly all patients with T2D are obese, the same processes likely contribute to the greater BMD these patients than in individuals with obesity but without diabetes. Further, BMD is positively associated with BMI [63], and skeletal mass adapts to the current mechanical demands, reflecting a physiological process. In addition, diabetes treatment may also modulate BMD [64]. Sulfonylureas and metformin reportedly have neutral or marginally protective relationships with fracture risk [64]. T1D is mediated by T lymphocytes [65], leading to autoimmune insulitis and characterized by selective islet beta cell damage. In contrast, T2D results from the interaction between genetic and environmental factors, giving rise to the development of a complex polygenic hereditary disease characterized by insulin resistance and defective islet beta cell function (insulin hyposecretion) [66].

This study has certain limitations. First, the relationship between diabetes and BMD/fracture may be confounded by the pleiotropic effect of diabetes-associated SNPs [67]. Therefore, our results may represent a shared genetic basis between diabetes and BMD/fracture rather than a causal relationship. Second, the GWAS summary data mostly included individuals of European ancestry, raising caution in generalizing our findings to populations with different racial and ethnic backgrounds. Third, due to insufficient sex-stratified GWAS summary data on BMD, we were unable to conduct separate analyses for men and women. Lastly, our MR analysis results were solely based on bioinformatics analyses, limiting our ability to elucidate the underlying mechanisms. Further confirmation of our findings would necessitate in vitro and in vivo investigations in future studies.

## Conclusions

Our study revealed that T1D and T2D have different effects on BMD at the genetic level. Additionally, BMD decreased in patients with T1D and increased in patients with T2D. These findings underscore the complex relationship between diabetes and bone health, highlighting the need for tailored interventions. Future research is warranted to elucidate underlying mechanisms and validate these associations across diverse populations, potentially informing targeted therapies and preventive strategies for individuals with diabetes.

### Electronic supplementary material

Below is the link to the electronic supplementary material.

Supplementary Material 1

## Data Availability

The datasets analyzed during the current study are available in the GWAS catalog (GWAS Catalog (ebi.ac.uk)). All data generated during this study are included in supplementary files.

## Declarations

### Transparency

The manuscript is an authentic, accurate, and transparent account of the study being reported, according to the lead authors (YQ/CX/ZZ). We also affirm that no significant aspects of the study have been left out and that any discrepancies from the study’s original design have been adequately explained.

## Abbreviations

BMD: Bone mineral density
OP: Osteoporosis
T1D: Type 1 diabetes
T2D: Type 2 diabetes
HbA1c: Glycosylated hemoglobin
MR: Mendelian randomization
FN: Femoral neck
LS: Lumbar spine
TB: Total body
BMI: Body mass index
IVW: Inverse variance weighted
MR-PRESSO: MR-Pleiotropy Residual Sum and Outlier
IVs: Instrumental variables
LD: Linkage disequilibrium
GWAS: Genome wide association study
SNPs: Single nucleotide polymorphisms

## References

[1] Global regional (2018). National disability-adjusted life-years (DALYs) for 359 diseases and injuries and healthy life expectancy (HALE) for 195 countries and territories, 1990–2017: a systematic analysis for the global burden of Disease Study 2017. Lancet (London England).

[2] Cho NH, Shaw JE, Karuranga S, Huang Y, da Rocha Fernandes JD, Ohlrogge AW, Malanda B (2018). IDF Diabetes Atlas: global estimates of diabetes prevalence for 2017 and projections for 2045. Diabetes Res Clin Pract.

[3] van Belle TL, Coppieters KT, von Herrath MG (2011). Type 1 diabetes: etiology, immunology, and therapeutic strategies. Physiol Rev.

[4] Khosla S, Samakkarnthai P, Monroe DG, Farr JN (2021). Update on the pathogenesis and treatment of skeletal fragility in type 2 diabetes mellitus. Nat Reviews Endocrinol.

[5] Morrish NJ, Wang SL, Stevens LK, Fuller JH, Keen H (2001). Mortality and causes of death in the WHO multinational study of Vascular Disease in Diabetes. Diabetologia.

[6] Ogurtsova K, da Rocha Fernandes JD, Huang Y, Linnenkamp U, Guariguata L, Cho NH, Cavan D, Shaw JE, Makaroff LE (2017). IDF Diabetes Atlas: global estimates for the prevalence of diabetes for 2015 and 2040. Diabetes Res Clin Pract.

[7] Johnston CB, Dagar M (2020). Osteoporosis in older adults. Med Clin N Am.

[8] Kanis JA, Oden A, Johnell O, Johansson H, De Laet C, Brown J, Burckhardt P, Cooper C, Christiansen C, Cummings S (2007). The use of clinical risk factors enhances the performance of BMD in the prediction of hip and osteoporotic fractures in men and women. Osteoporos International: J Established as Result Cooperation between Eur Foundation Osteoporos Natl Osteoporos Foundation USA.

[9] Johnell O, Kanis JA (2006). An estimate of the worldwide prevalence and disability associated with osteoporotic fractures. Osteoporos International: J Established as Result Cooperation between Eur Foundation Osteoporos Natl Osteoporos Foundation USA.

[10] Lane NE (2006). Epidemiology, etiology, and diagnosis of osteoporosis. Am J Obstet Gynecol.

[11] Salari N, Ghasemi H, Mohammadi L, Behzadi MH, Rabieenia E, Shohaimi S, Mohammadi M (2021). The global prevalence of osteoporosis in the world: a comprehensive systematic review and meta-analysis. J Orthop Surg Res.

[12] Abdulameer SA, Sulaiman SA, Hassali MA, Subramaniam K, Sahib MN (2012). Osteoporosis and type 2 diabetes mellitus: what do we know, and what we can do?. Patient Prefer Adherence.

[13] Gunczler P, Lanes R, Paoli M, Martinis R, Villaroel O, Weisinger JR (2001). Decreased bone mineral density and bone formation markers shortly after diagnosis of clinical type 1 diabetes mellitus. J Pediatr Endocrinol Metabolism: JPEM.

[14] Rehling T, Bjørkman AD, Andersen MB, Ekholm O, Molsted S. Diabetes Is Associated with Musculoskeletal Pain, Osteoarthritis, Osteoporosis, and Rheumatoid Arthritis. *Journal of diabetes research* 2019, 2019:6324348. 10.1155/2019/6324348 PMC6925775 31886282

[15] Rakic V, Davis WA, Chubb SA, Islam FM, Prince RL, Davis TM (2006). Bone mineral density and its determinants in diabetes: the Fremantle Diabetes Study. Diabetologia.

[16] Botushanov NP, Orbetzova MM (2009). Bone mineral density and fracture risk in patients with type 1 and type 2 diabetes mellitus. Folia Medica.

[17] Olmos JM, Pérez-Castrillón JL, García MT, Garrido JC, Amado JA, González-Macías J (1994). Bone densitometry and biochemical bone remodeling markers in type 1 diabetes mellitus. Bone Miner.

[18] Nicodemus KK, Folsom AR (2001). Type 1 and type 2 diabetes and incident hip fractures in postmenopausal women. Diabetes Care.

[19] Forsén L, Meyer HE, Midthjell K, Edna TH (1999). Diabetes mellitus and the incidence of hip fracture: results from the Nord-Trøndelag Health Survey. Diabetologia.

[20] Heath H, Melton LJ, Chu CP (1980). Diabetes mellitus and risk of skeletal fracture. N Engl J Med.

[21] Melchior TM, Sørensen H, Torp-Pedersen C (1994). Hip and distal arm fracture rates in peri- and postmenopausal insulin-treated diabetic females. J Intern Med.

[22] Davey Smith G, Hemani G (2014). Mendelian randomization: genetic anchors for causal inference in epidemiological studies. Hum Mol Genet.

[23] Fan J, Zhu J, Sun L, Li Y, Wang T, Li Y (2021). Causal association of adipokines with osteoarthritis: a mendelian randomization study. Rheumatology (Oxford).

[24] ElSayed NA, Aleppo G, Aroda VR, Bannuru RR, Brown FM, Bruemmer D, Collins BS, Hilliard ME, Isaacs D, Johnson EL (2023). 2. Classification and diagnosis of diabetes: standards of Care in Diabetes-2023. Diabetes Care.

[25] Davies MJ, Aroda VR, Collins BS, Gabbay RA, Green J, Maruthur NM, Rosas SE, Del Prato S, Mathieu C, Mingrone G (2022). Management of hyperglycemia in type 2 diabetes, 2022. A Consensus Report by the American Diabetes Association (ADA) and the European Association for the Study of Diabetes (EASD). Diabetes Care.

[26] Skrivankova VW, Richmond RC, Woolf BAR, Yarmolinsky J, Davies NM, Swanson SA, VanderWeele TJ, Higgins JPT, Timpson NJ, Dimou N (2021). Strengthening the reporting of Observational studies in Epidemiology using mendelian randomization: the STROBE-MR Statement. JAMA.

[27] Censin JC, Nowak C, Cooper N, Bergsten P, Todd JA, Fall T (2017). Childhood adiposity and risk of type 1 diabetes: a mendelian randomization study. PLoS Med.

[28] Zhao W, Rasheed A, Tikkanen E, Lee JJ, Butterworth AS, Howson JMM, Assimes TL, Chowdhury R, Orho-Melander M, Damrauer S (2017). Identification of new susceptibility loci for type 2 diabetes and shared etiological pathways with coronary heart disease. Nat Genet.

[29] Soranzo N, Sanna S, Wheeler E, Gieger C, Radke D, Dupuis J, Bouatia-Naji N, Langenberg C, Prokopenko I, Stolerman E (2010). Common variants at 10 genomic loci influence hemoglobin A_1_(C) levels via glycemic and nonglycemic pathways. Diabetes.

[30] Estrada K, Styrkarsdottir U, Evangelou E, Hsu YH, Duncan EL, Ntzani EE, Oei L, Albagha OM, Amin N, Kemp JP (2012). Genome-wide meta-analysis identifies 56 bone mineral density loci and reveals 14 loci associated with risk of fracture. Nat Genet.

[31] Medina-Gomez C, Kemp JP, Trajanoska K, Luan J, Chesi A, Ahluwalia TS, Mook-Kanamori DO, Ham A, Hartwig FP, Evans DS (2018). Life-Course Genome-Wide Association Study Meta-Analysis of Total Body BMD and Assessment of Age-Specific effects. Am J Hum Genet.

[32] Morris JA, Kemp JP, Youlten SE, Laurent L, Logan JG, Chai RC, Vulpescu NA, Forgetta V, Kleinman A, Mohanty ST (2019). An atlas of genetic influences on osteoporosis in humans and mice. Nat Genet.

[33] Wang Y, Li T, Fu L, Yang S, Hu YQ (2021). A Novel Method for mendelian randomization analyses with pleiotropy and linkage disequilibrium in genetic variants from Individual Data. Front Genet.

[34] Johnell O, Kanis JA, Oden A, Johansson H, De Laet C, Delmas P, Eisman JA, Fujiwara S, Kroger H, Mellstrom D (2005). Predictive value of BMD for hip and other fractures. J bone Mineral Research: Official J Am Soc Bone Mineral Res.

[35] Zheng HF, Forgetta V, Hsu YH, Estrada K, Rosello-Diez A, Leo PJ, Dahia CL, Park-Min KH, Tobias JH, Kooperberg C (2015). Whole-genome sequencing identifies EN1 as a determinant of bone density and fracture. Nature.

[36] Bowden J, Davey Smith G, Haycock PC, Burgess S (2016). Consistent estimation in mendelian randomization with some Invalid instruments using a weighted median estimator. Genet Epidemiol.

[37] Arnold M, Raffler J, Pfeufer A, Suhre K, Kastenmüller G (2015). SNiPA: an interactive, genetic variant-centered annotation browser. Bioinf (Oxford England).

[38] Lanzinger S, Zimmermann A, Ranjan AG, Gani O, Pons Perez S, Akesson K, Majidi S, Witsch M, Hofer S, Johnson S et al. A collaborative comparison of international pediatric diabetes registries. *Pediatric diabetes* 2022. 10.1111/pedi.13362 35561091

[39] Baxter-Jones AD, Faulkner RA, Forwood MR, Mirwald RL, Bailey DA (2011). Bone mineral accrual from 8 to 30 years of age: an estimation of peak bone mass. J bone Mineral Research: Official J Am Soc Bone Mineral Res.

[40] Chen SC, Shepherd S, McMillan M, McNeilly J, Foster J, Wong SC, Robertson KJ, Ahmed SF (2019). Skeletal fragility and its clinical determinants in children with type 1 diabetes. J Clin Endocrinol Metab.

[41] Syversen U, Mosti MP, Mynarek IM, Vedal TSJ, Aasarød K, Basso T, Reseland JE, Thorsby PM, Asvold BO, Eriksen EF (2021). Evidence of impaired bone quality in men with type 1 diabetes: a cross-sectional study. Endocr Connections.

[42] Napoli N, Chandran M, Pierroz DD, Abrahamsen B, Schwartz AV, Ferrari SL (2017). Mechanisms of diabetes mellitus-induced bone fragility. Nat Reviews Endocrinol.

[43] Tang Y, Zhang L, Ye D, Zhao A, Liu Y, Zhang M (2023). Causal relationship between type 1 diabetes and osteoporosis and fracture occurrence: a two-sample mendelian randomization analysis. Osteoporos International: J Established as Result Cooperation between Eur Foundation Osteoporos Natl Osteoporos Foundation USA.

[44] Weber DR, Schwartz G (2016). Epidemiology of Skeletal Health in type 1 diabetes. Curr Osteoporos Rep.

[45] McComb C, Harpur A, Yacoubian C, Leddy C, Anderson G, Shepherd S, Perry C, Shaikh MG, Foster J, Ahmed SF (2014). MRI-based abnormalities in young adults at risk of adverse bone health due to childhood-onset metabolic & endocrine conditions. Clin Endocrinol.

[46] Cheng L, Wang S, Tang H (2023). Type 2 diabetes mellitus plays a protective role against osteoporosis --mendelian randomization analysis. BMC Musculoskelet Disord.

[47] Ahmad OS, Leong A, Miller JA, Morris JA, Forgetta V, Mujammami M, Richards JB (2017). A mendelian randomization study of the effect of Type-2 diabetes and glycemic traits on bone Mineral Density. J bone Mineral Research: Official J Am Soc Bone Mineral Res.

[48] Kindler JM, Gallo S, Khoury PR, Urbina EM, Zemel BS. Diet Quality and Bone Density in Youth with Healthy Weight, obesity, and type 2 diabetes. Nutrients 2021, 13(9). 10.3390/nu13093288 PMC8472061 34579165

[49] de Klift LII, de Laet M, van Daele CE, Hofman PL, Pols A (2005). Bone mineral density and fracture risk in type-2 diabetes mellitus: the Rotterdam Study. Osteoporos International: J Established as Result Cooperation between Eur Foundation Osteoporos Natl Osteoporos Foundation USA.

[50] Gatling W, Guzder RN, Turnbull JC, Budd S, Mullee MA (2001). The Poole Diabetes Study: how many cases of type 2 diabetes are diagnosed each year during normal health care in a defined community?. Diabetes Res Clin Pract.

[51] Chen H, Zhou X, Fujita H, Onozuka M, Kubo KY (2013). Age-related changes in trabecular and cortical bone microstructure. Int J Endocrinol.

[52] Thrailkill KM, Lumpkin CK, Bunn RC, Kemp SF, Fowlkes JL (2005). Is insulin an anabolic agent in bone? Dissecting the diabetic bone for clues. Am J Physiol Endocrinol Metabolism.

[53] Hamann C, Kirschner S, Günther KP, Hofbauer LC (2012). Bone, sweet bone–osteoporotic fractures in diabetes mellitus. Nat Reviews Endocrinol.

[54] Patsch JM, Burghardt AJ, Yap SP, Baum T, Schwartz AV, Joseph GB, Link TM (2013). Increased cortical porosity in type 2 diabetic postmenopausal women with fragility fractures. J bone Mineral Research: Official J Am Soc Bone Mineral Res.

[55] Botella Martínez S, Varo Cenarruzabeitia N, Escalada San Martin J, Calleja Canelas A (2016). The diabetic paradox: bone mineral density and fracture in type 2 diabetes. Endocrinologia Y Nutricion: organo de la Sociedad Esp De Endocrinologia Y Nutricion.

[56] Li G, Prior JC, Leslie WD, Thabane L, Papaioannou A, Josse RG, Kaiser SM, Kovacs CS, Anastassiades T, Towheed T (2019). Frailty and Risk of fractures in patients with type 2 diabetes. Diabetes Care.

[57] Kennedy CC, Ioannidis G, Rockwood K, Thabane L, Adachi JD, Kirkland S, Pickard LE, Papaioannou A (2014). A Frailty Index predicts 10-year fracture risk in adults age 25 years and older: results from the Canadian Multicentre osteoporosis study (CaMos). Osteoporos International: J Established as Result Cooperation between Eur Foundation Osteoporos Natl Osteoporos Foundation USA.

[58] Joad S, Ballato E, Deepika F, Gregori G, Fleires-Gutierrez AL, Colleluori G, Aguirre L, Chen R, Russo V, Fuenmayor Lopez VC (2021). Hemoglobin A1c threshold for reduction in bone turnover in men with type 2 diabetes Mellitus. Front Endocrinol.

[59] Thong EP, Herath M, Weber DR, Ranasinha S, Ebeling PR, Milat F, Teede H (2018). Fracture risk in young and middle-aged adults with type 1 diabetes mellitus: a systematic review and meta-analysis. Clin Endocrinol.

[60] Dytfeld J, Michalak M (2017). Type 2 diabetes and risk of low-energy fractures in postmenopausal women: meta-analysis of observational studies. Aging Clin Exp Res.

[61] Fan Y, Wei F, Lang Y, Liu Y (2016). Diabetes mellitus and risk of hip fractures: a meta-analysis. Osteoporos International: J Established as Result Cooperation between Eur Foundation Osteoporos Natl Osteoporos Foundation USA.

[62] Vestergaard P (2007). Discrepancies in bone mineral density and fracture risk in patients with type 1 and type 2 diabetes–a meta-analysis. Osteoporos International: J Established as Result Cooperation between Eur Foundation Osteoporos Natl Osteoporos Foundation USA.

[63] De Laet C, Kanis JA, Odén A, Johanson H, Johnell O, Delmas P, Eisman JA, Kroger H, Fujiwara S, Garnero P (2005). Body mass index as a predictor of fracture risk: a meta-analysis. Osteoporos International: J Established as Result Cooperation between Eur Foundation Osteoporos Natl Osteoporos Foundation USA.

[64] Palermo A, D’Onofrio L, Eastell R, Schwartz AV, Pozzilli P, Napoli N (2015). Oral anti-diabetic drugs and fracture risk, cut to the bone: safe or dangerous? A narrative review. Osteoporos International: J Established as Result Cooperation between Eur Foundation Osteoporos Natl Osteoporos Foundation USA.

[65] de Jong VM, van der Slik AR, Laban S, van ’t Slot R, Koeleman BP, Zaldumbide A, Roep BO (2016). Survival of autoreactive T lymphocytes by microRNA-mediated regulation of apoptosis through TRAIL and Fas in type 1 diabetes. Genes Immun.

[66] Prasad RB, Groop L (2015). Genetics of type 2 diabetes-pitfalls and possibilities. Genes.

[67] Prudente S, Trischitta V (2006). Editorial: the pleiotropic effect of the ENPP1 (PC-1) gene on insulin resistance, obesity, and type 2 diabetes. J Clin Endocrinol Metab.

