# Supplementary material for: Diabetes and osteoporosis: a two-sample mendelian randomization study
Source: BMC Musculoskelet Disord. 2024 Apr 23;25:317. doi: 10.1186/s12891-024-07430-0 (PMC11036742; doi:10.1186/s12891-024-07430-0)
Supplement: Supplementary file 1 — Supplementary Material 1 [file 12891_2024_7430_MOESM1_ESM.pdf]

Supplementary Tables

Table S1. Mendelian Randomization estimates of the effect of T1D risk on bone mineral density and fracture.

				MR results		Sensitivity analysis		
						Pleiotropy test	Heterogeneity tests	
**Exposure**	**Outcome**	**No. of IVs**	**Method**	***β* (95%CI)**	***P*-value**	**MR-Egger intercept (*P*)**	**Cochran's Q (*P*) IVW MR-Egger**	**random effects mode (IVW)**
T1D	FN-BMD	47	MR-Egger	0.011 (-0.008, 0.031)	0.264	-0.002 (0.490)	66.426 (**0.021**) 67.140 (**0.023**)	0.368
			Weighted median	0.010 (-0.007, 0.027)	0.246			
			IVW	0.007 (-0.008, 0.021)	0.368			
			Simple mode	0.049 (-0.009, 0.103)	0.105			
			Weighted mode	0.014 (-0.001, 0.029)	0.077			
			MR-PRESSO		0.783			
	LS-BMD	47	MR-Egger	0.002 (-0.020, 0.024)	0.854	0.003 (0.268)	58.256 (0.089) 59.881 (0.082)	---
			Weighted median	0.001 (-0.018, 0.020)	0.911			
			IVW	0.010 (-0.006, 0.026)	0.205			
			Simple mode	-0.016 (-0.066, 0.034)	0.532			
			Weighted mode	-0.001 (-0.020, 0.018)	0.926			
			MR-PRESSO		0.077			

		**MR-Egger**	0.001 (-0.006, 0.009)	0.770		81.912 (**7.848e-06**)	
		**Weighted median**	0.001 (-0.005, 0.005)	0.936			
**Heel-BMD**	**36**	**IVW**	0.002 (-0.004, 0.007)	0.561	0.001 (0.866)		0.561
		**Simple mode**	0.010 (-0.006,0.026)	0.226		81.981 (**1.224e-05**)	
		**Weighted mode**	0.002 (-0.003, 0.006)	0.513			
		**MR-PRESSO**		0.972			
		**MR-Egger**	-0.021 (-0.038, -0.005)	**0.015**			
		**Weighted median**	-0.023 (-0.037, -0.008)	**0.002**		58.128 (0.075)	
**TB-BMD**	**46**	**IVW**	-0.018 (-0.030, -0.006)	**0.002**	0.001 (0.591)		---
		**Simple mode**	-0.011 (-0.051, 0.030)	0.612			
		**Weighted mode**	-0.021 (-0.037, -0.006)	**0.010**		58.516 (0.085)	
		**MR-PRESSO**		0.469			
		**MR-Egger**	-0.001 (-0.015, 0.014)	0.945			
		**Weighted median**	0.001 (-0.012, 0.015)	0.832		59.342 (0.061)	
**Fracture**	**46**	**IVW**	-0.001 (-0.012, 0.010)	0.824	-0.001 (0.882)		---
		**Simple mode**	-0.024 (-0.064, 0.017)	0.253			
		**Weighted mode**	-0.001 (-0.014, 0.012)	0.914		59.373 (0.074)	
		**MR-PRESSO**		0.130			

T1D: type 1 diabetes; FN: femoral neck; LS: lumbar spine; TB: total body; IVW: inverse-variance weighted; IVs: instrumental variables; MR-PRESSO: Mendelian Randomization Pleiotropy Residual Sum and Outlier.

Table S2. Mendelian Randomization estimates of the effect of T2D risk on bone mineral density and fracture.

Exposure	Outcome	No. of IVs	Method	MR results		Sensitivity analysis		
						Pleiotropy test	Heterogeneity tests	
				β (SE)	*P*-value	MR-Egger intercept (*P*)	Cochran's Q (*P*)	random effects mode (IVW)
T2D	FN-BMD	55	**MR-Egger**	0.029 (-0.035, 0.092)	0.379	0.001 (0.888)	64.068 (0.142)	---
			**Weighted median**	0.042 (-0.006, 0.090)	0.085			
			**IVW**	0.033 (0.003, 0.062)	**0.030**		64.092 (0.164)	
			**Simple mode**	0.060 (-0.032, 0.152)	0.209			
			**Weighted mode**	0.053 (0.005, 0.100)	**0.034**			
			**MR-PRESSO**		0.783			
	LS-BMD	55	**MR-Egger**	-0.073 (-0.200, 0.053)	0.262	0.009 (0.082)	70.711 (0.052)	0.109
			**Weighted median**	0.043 (-0.011, 0.098)	0.119			
			**IVW**	0.035 (-0.008, 0.077)	0.109		70.900 (0.031)	
			**Simple mode**	0.045 (-0.062, 0.152)	0.415			
			**Weighted mode**	0.048 (-0.044, 0.139)	0.312			
			**MR-PRESSO**		0.077			
	Heel-BMD	41	**MR-Egger**	0.029 (0.004, 0.054)	**0.030**	-0.001 (0.346)	116.318 (**1.276e-09**)	**0.004**
			**Weighted median**	0.032 (0.019, 0.045)	**1.443 e-06**			

		**IVW**	0.018 (0.006, 0.031)	**0.004**		119.029	
		**Simple mode**	-0.021 (-0.060, 0.019)	0.309		(**8.876e-10**)	
		**Weighted mode**	0.033 (0.019, 0.046)	2.966			
		**MR-PRESSO**		0.972			
		**MR-Egger**	0.051 (-0.011, 0.112)	0.111		95.996	
		**Weighted median**	0.055 (0.018, 0.092)	**0.003**		(**9.929e-05**)	
**TB-BMD**	**52**	**IVW**	0.050 (0.022, 0.079)	**0.001**	-5.119e-05		**0.001**
		**Simple mode**	0.055 (-0.012, 0.123)	0.114	(0.985)	95.997	
		**Weighted mode**	0.059 (0.024, 0.095)	**0.002**		(**0.001**)	
		**MR-PRESSO**		0.469			
		**MR-Egger**	0.029 (-0.036, 0.093)	0.038		97.724	
		**Weighted median**	0.034 (-0.001, 0.070)	0.059		(0.001)	
**Fracture**	**57**	**IVW**	-0.004 (-0.034, 0.026)	0.794	-0.003		0.794
		**Simple mode**	0.016 (-0.058, 0.090)	0.671	(0.264)	99.986	
		**Weighted mode**	0.030 (-0.010, 0.070)	0.148		(0.001)	
		**MR-PRESSO**		0.130			

T2D: type 2 diabetes; FN: femoral neck; LS: lumbar spine; TB: total body; IVW: inverse-variance weighted; IVs: instrumental variables; MR-PRESSO: Mendelian Randomization Pleiotropy Residual Sum and Outlier.

Table S3. Mendelian Randomization estimates of the effect of HbA1c risk on bone mineral density and fracture.

Exposure	Outcome	No. of IVs	Method	MR results: *β* (SE)	MR results: *P*-value	Sensitivity analysis: Pleiotropy test: MR-Egger intercept (*P*)	Sensitivity analysis: Heterogeneity tests: Cochran's Q (*P*)	Sensitivity analysis: Heterogeneity tests: random effects mode (IVW)
HbA1c	FN-BMD	10	MR-Egger	-0.183 (-0.537, 0.171)	0.341	0.009 (0.273)	7.498 (0.484)	---
			Weighted median	-0.046 (-0.238, 0.146)	0.640			
			IVW	0.012 (-0.131, 0.154)	0.873		8.883 (0.448)	
			Simple mode	-0.031 (-0.322, 0.259)	0.837			
			Weighted mode	-0.045 (-0.295, 0.205)	0.731			
			MR-PRESSO		0.783			
	LS-BMD	10	MR-Egger	-0.058 (-0.470, 0.354)	0.789	0.006 (0.510)	5.830 (0.666)	---
			Weighted median	0.042 (-0.170, 0.255)	0.695			
			IVW	0.075 (-0.091, 0.241)	0.377		6.306 (0.709)	
			Simple mode	0.002 (-0.317, 0.320)	0.992			
			Weighted mode	0.021 (-0.235, 0.278)	0.874			
			MR-PRESSO		0.077			
	Heel-BMD	10	MR-Egger	-0.045 (-0.268, 0.178)	0.702	0.002 (0.683)	56.109 (**2.687e-09**)	0.982
			Weighted median	-0.067 (-0.125, -0.009)	0.025			
			IVW	-0.001 (-0.087, 0.085)	0.982			

		**Simple mode**	-0.067 (-0.154, 0.019)	0.187		57.369	
		**Weighted mode**	-0.067 (-0.123, -0.012)	0.046		(**4.295e-09**)	
		**MR-PRESSO**		0.972			
		**MR-Egger**	-0.118 (-0.388, 0.152)	0.418		3.285	
		**Weighted median**	0.030 (-0.111, 0.172)	0.676		(0.915)	
**TB-BMD**	**10**	**IVW**	0.042 (-0.065, 0.150)	0.441	0.007		---
		**Simple mode**	0.040 (-0.189, 0.270)	0.738	(0.241)	4.889	
		**Weighted mode**	-0.028 (-0.224, 0.169)	0.789		(0.844)	
		**MR-PRESSO**		0.469			
		**MR-Egger**	-0.015 (-0.313, 0.284)	0.927		5.227	
		**Weighted median**	0.017 (-0.135, 0.169)	0.829		(0.733)	
**Fracture**	**10**	**IVW**	-0.012 (-0.133, 0.109)	0.843	0.001		---
		**Simple mode**	0.050 (-0.168, 0.268)	0.663	(0.987)	5.228	
		**Weighted mode**	0.008 (-0.172, 0.188)	0.933		(0.814)	
		**MR-PRESSO**		0.130			

HbA1c: glycosylated hemoglobin; FN: femoral neck; LS: lumbar spine; TB: total body; IVW: inverse-variance weighted; IVs: instrumental variables; MR-PRESSO: Mendelian Randomization Pleiotropy Residual Sum and Outlier.

Table S4. Subgroup analysis using Mendelian Randomization estimates for T1D on total-body bone mineral density by age.

Exposure	Outcome	No. of IVs	Method	MR results: β (SE)	MR results: P-value	Sensitivity analysis: Pleiotropy test: MR-Egger intercept (P)	Sensitivity analysis: Heterogeneity tests: Cochran's Q (P)	Sensitivity analysis: Heterogeneity tests: random effects mode (IVW)
T1D	TB-BMD (0~15 Y)	47	MR-Egger	-0.011 (-0.045, 0.023)	0.512	0.004 (0.254)	52.424 (0.208)	---
			Weighted median	-0.026 (-0.059, 0.007)	0.124			
			IVW	0.003 (-0.021, 0.027)	0.810		53.982 (0.196)	
			Simple mode	-0.024 (-0.104, 0.056)	0.556			
			Weighted mode	-0.022 (-0.055, 0.011)	0.189			
			MR-PRESSO		0.186			
	TB-BMD (15~30 Y)	47	MR-Egger	-0.001 (-0.081, 0.079)	0.980	-0.001 (0.922)	59.610 (0.071)	---
			Weighted median	-0.014 (-0.090, 0.061)	0.707			
			IVW	-0.004 (-0.055, 0.047)	0.875		59.623 (0.086)	
			Simple mode	-0.009 (-0.166, 0.148)	0.912			
			Weighted mode	0.009 (-0.061, 0.078)	0.804			
			MR-PRESSO		0.170			
	TB-BMD (30~45 Y)	47	MR-Egger	-0.001 (-0.035, 0.032)	0.936	-0.001 (0.844)	38.194 (0.754)	---
			Weighted median	-0.008 (-0.043, 0.028)	0.668			

		**IVW**	-0.004 (-0.028, 0.020)	0.764			
		**Simple mode**	-0.015 (-0.069, 0.099)	0.721		38.234	
		**Weighted mode**	-0.003 (-0.036, 0.029)	0.847		(0.785)	
		**MR-PRESSO**		0.767			
		**MR-Egger**	-0.011 (-0.044, 0.023)	0.538		86.197	
		**Weighted median**	-0.027 (-0.052, -0.002)	**0.036**		(**0.001**)	
**TB-BMD**		**IVW**	-0.004 (-0.028, 0.020)	0.761	0.002		
**(45~60 Y)**	**47**	**Simple mode**	-0.013 (-0.094, 0.068)	0.754	(0.564)	86.846	0.761
		**Weighted mode**	-0.023 (-0.050, 0.004)	0.096		(**0.001**)	
		**MR-PRESSO**		0.576			
		**MR-Egger**	-0.003 (-0.034, 0.028)	0.851		73.544	
		**Weighted median**	-0.023 (-0.048, 0.002)	0.076		(**0.005**)	
**TB-BMD**		**IVW**	-0.006 (-0.028, 0.015)	0.568	-0.001 (0.		
**(＞60 Y)**	**47**	**Simple mode**	-0.019 (-0.094, 0.055)	0.614	774)	73.681	0.568
		**Weighted mode**	-0.021 (-0.047, 0.005)	0.119		(0.006)	
		**MR-PRESSO**		0.486			

T1D: type 1 diabetes; TB: total body; IVW: inverse-variance weighted; IVs: instrumental variables; MR-PRESSO: Mendelian Randomization Pleiotropy Residual Sum and Outlier.

Table S5. Subgroup analysis using Mendelian Randomization estimates for T2D on total-body bone mineral density by age.

Exposure	Outcome	No. of IVs	Method	MR results		Sensitivity analysis		
						Pleiotropy test	Heterogeneity tests	
				β (SE)	*P*-value	MR-Egger intercept (*P*)	Cochran's Q (*P*)	random effects mode (IVW)
T2D	TB-BMD (0~15 Y)	57	**MR-Egger**	0.057 (-0.047, 0.161)	0.291			
			**Weighted median**	0.076 (-0.001, 0.152)	0.051		63.748 (0.196)	
			**IVW**	0.058 (0.009, 0.106)	**0.019**	0.001 (0.980)		---
			**Simple mode**	0.014 (-0.139, 0.168)	0.855			
			**Weighted mode**	0.040 (-0.043, 0.123)	0.348		63.748 (0.223)	
			**MR-PRESSO**		0.128			
	TB-BMD (15~30 Y)	57	**MR-Egger**	0.084 (-0.107, 0.275)	0.393			
			**Weighted median**	0.065 (-0.080, 0.209)	0.382		69.006 (0.097)	
			**IVW**	0.068 (-0.020, 0.157)	0.128	-0.001 (0.859)		---
			**Simple mode**	0.121 (-0.199, 0.441)	0.461			
			**Weighted mode**	0.081 (-0.079, 0.242)	0.325		69.046 (0.113)	
			**MR-PRESSO**		0.118			
	TB-BMD (30~45 Y)	57	**MR-Egger**	0.040 (-0.088, 0.168	0.542			
			**Weighted median**	0.028 (-0.060, 0.116)	0.530	0.001 (0.971)	74.879 (**0.039**)	0.165
			**IVW**	0.042 (-0.017, 0.102)	0.165			

		**Simple mode**	0.034 (-0.158, 0.226)	0.732		74.881 (**0.047**)	
		**Weighted mode**	0.030 (-0.061, 0.122)	0.520			
		**MR-PRESSO**		0.057			
**TB-BMD (45~60 Y)**	**57**	**MR-Egger**	-0.038 (-0.131, 0.055	0.428			
		**Weighted median**	0.019 (-0.050, 0.088)	0.588		75.690 (**0.034**)	
		**IVW**	0.038 (-0.007, 0.082)	0.099	0.007 (0.077)		0.099
		**Simple mode**	0.020 (-0.113, 0.153)	0.770			
		**Weighted mode**	0.009 (-0.066, 0.083)	0.819		80.160 (**0.019**)	
		**MR-PRESSO**		0.558			
**TB-BMD (＞60 Y)**	**57**	**MR-Egger**	0.032 (-0.071, 0.134)	0.545			
		**Weighted median**	0.106 (0.052, 0.161)	**0.001**		110.107 (**1.496e-05**)	
		**IVW**	0.065 (0.017, 0.113)	**0.008**	0.003 (0.473)		**0.008**
		**Simple mode**	0.122 (-0.015, 0.259)	0.087			
		**Weighted mode**	0.105 (0.037, 0.173)	**0.004**		111.154 (**1.639e-05**)	
		**MR-PRESSO**		0.836			

T2D: type 2 diabetes; TB: total body; IVW: inverse-variance weighted; IVs: instrumental variables; MR-PRESSO: Mendelian Randomization Pleiotropy Residual Sum and Outlier.

Table S6. Results of two-sample Mendelian randomization analyses of type 1 diabetes on bone mineral density/fracture.

SNP	Effect allele/ Other allele	SNP & Type 1 diabetes		SNP & bone mineral density associations									
				FN-BMD		LS-BMD		Heel-BMD		TB-BMD		Fracture	
		Beta (SE)	*P*	Beta (SE)	*P*	Beta (SE)	*P*	Beta (SE)	*P*	Beta (SE)	*P*	Beta (SE)	*P*
**Type 1 diabetes**													
rs1004707	T/C	0.107(0.019)	4.31e-08	-0.089 (0.085)	0.297	-0.087(0.098)	0.378	0.005(0.019)	0.800	0.090(0.065)	0.164	-0.053(0.074)	0.473
rs10214237	C/T	0.107(0.016)	2.32e-11	-0.091(0.079)	0.247	0.031(0.092)	0.739	---	---	0.043(0.060)	0.472	-0.048(0.068)	0.485
rs1022674	A/C	0.100(0.018)	2.20e-08	0.056(0.081)	0.490	0.070(0.095)	0.463	0.012(0.020)	0.547	0.043(0.061)	0.479	-0.114(0.072)	0.112
rs1048055	A/C	0.110(0.016)	1.03e-11	-0.173(0.073)	0.019	-0.037(0.085)	0.667	0.012(0.018)	0.513	0.032(0.056)	0.572	0.014(0.063)	0.826
rs11066320	A/G	0.179(0.015)	1.88e-33	-0.158(0.045)	0.000	---	---	---	---	---	---	---	---
rs11085725	T/C	0.126(0.017)	5.86e-14	-0.092(0.067)	0.173	-0.056(0.078)	0.476	-0.049(0.016)	0.003	-0.053(0.052)	0.303	0.109(0.057)	0.056
rs11203203	A/G	0.129(0.015)	3.25e-17	0.048(0.060)	0.426	0.095(0.070)	0.173	-0.029(0.015)	0.051	0.010(0.045)	0.823	0.067(0.053)	0.208
rs114607072	T/G	1.124(0.067)	5.78e-63	0.026(0.081)	0.375	0.009(0.022)	0.685	---	---	0.015(0.016)	0.370	-0.024(0.015)	0.102
rs11978267	G/A	0.089(0.016)	3.10e-08	7.826(0.093)	0.999	-0.012(0.109)	0.915	0.025(0.023)	0.266	-0.083(0.072)	0.248	-0.120(0.082)	0.142
rs12506688	T/C	0.101(0.016)	1.44e-10	-0.097(0.081)	0.235	-0.017(0.095)	0.853	0.020(0.020)	0.305	-0.048(0.061)	0.429	-0.038(0.070)	0.591
rs1264813	T/C	0.336(0.023)	8.88e-49	-0.059(0.050)	0.239	-0.089(0.067)	0.181	---	---	-0.009(0.034)	0.801	-0.008(0.033)	0.816
rs12927355	T/C	0.155(0.016)	7.29e-23	-0.023(0.052)	0.662	-0.026(0.062)	0.663	---	---	0.022(0.039)	0.577	-0.054(0.045)	0.229
rs12979891	C/T	0.094(0.015)	2.25e-10	0.107(0.080)	0.181	0.253(0.094)	0.007	0.038(0.020)	0.051	---	---	0.030(0.070)	0.667
rs1352312	G/T	0.135(0.021)	6.60e-11	0.0690(0.105)	0.510	0.084(0.129)	0.512	---	---	-0.003(0.049)	0.952	---	---
rs151233	T/C	0.164(0.024)	7.46e-12	0.0160(0.071)	0.821	0.148(0.077)	(0.056)	0.004(0.017)	0.802	0.171(0.065)	0.008	-0.004(0.059)	0.950
rs1574285	G/T	0.103(0.014)	1.69e-12	0.141(0.075)	0.060	0.117(0.087)	0.181	0.004(0.018)	0.821	0.023(0.056)	0.679	-0.064(0.065)	0.325
rs1615504	T/C	0.103(0.015)	1.22e-12	0.074(0.072)	0.307	0.188(0.084)	0.026	-0.024(0.018)	0.192	0.135(0.055)	0.015	0.0462(0.063)	0.467
rs17652674	A/C	0.100(0.018)	2.20e-08	-0.010(0.095)	0.914	0.003(0.110)	0.978	-0.038(0.024)	0.110	-0.100(0.072)	0.165	0.070(0.083)	0.399
rs1990760	C/T	0.119(0.015)	1.83e-15	0.013(0.063)	0.831	-0.018(0.073)	0.804	-8.471(0.016)	0.996	-0.043(0.049)	0.379	-0.017(0.056)	0.756
rs1994565	G/T	0.096(0.017)	1.84e-08	-0.021(0.092)	0.816	-0.127(0.101)	0.206	0.006(0.021)	0.771	-0.056(0.068)	0.406	-0.076(0.075)	0.308

rs2212434	T/C	0.080(0.015)	3.54e-08	0.184(0.094)	0.050	0.286(0.109)	0.009	0.030(0.023)	0.195	0.0486(0.071)	(0.494)	-0.041(0.082)	0.619
rs2289702	T/C	0.171(0.026)	7.08e-11	0.117(0.073)	0.111	0.024(0.085)	0.775	0.040(0.0178)	0.023	-0.029(0.063)	0.650	-0.040(0.063)	0.521
rs2313430	C/T	0.085(0.015)	5.75e-09	0.104(0.092)	0.260	0.143(0.108)	0.187	-0.030(0.022)	0.173	0.007(0.068)	0.918	-0.018(0.077)	0.812
rs2476601	A/G	0.544(0.022)	4.54e-135	0.055(0.023)	0.014	0.086(0.026)	0.001	0.011(0.006)	0.058	---	---	-0.024(0.020)	0.228
rs3024505	A/G	0.124(0.021)	1.80e-09	0.076(0.086)	0.376	-0.031(0.096)	0.744	-0.017(0.020)	0.415	-0.040(0.063)	0.522	0.013(0.073)	0.854
rs3087243	A/G	0.148(0.015)	3.70e-23	0.073(0.052)	0.160	0.036(0.060)	0.553	---	---	0.026(0.038)	0.505	0.022(0.044)	0.624
rs3129889	G/A	1.341(0.043)	3.41e-217	0.009(0.009)	0.314	0.001(0.010)	0.953	---	---	-0.024(0.008)	0.002	0.004(0.007)	0.559
rs36104352	C/A	0.155(0.025)	6.17e-10	-0.085(0.076)	0.263	0.077(0.088)	0.383	-0.053(0.018)	0.004	-0.069(0.060)	0.250	0.080(0.065)	0.215
rs434943	A/G	0.099(0.017)	8.22e-09	0.071(0.084)	0.404	-0.033(0.099)	0.740	-0.016(0.020)	0.411	0.05(0.065)	0.435	-0.106(0.070)	0.130
rs45485691	A/G	0.107(0.016)	5.53e-12	-0.068(0.080)	0.392	-0.072(0.093)	0.440	-0.014(0.019)	0.445	-0.065(0.061)	0.288	-0.067(0.069)	0.328
rs4804000	A/G	0.131(0.019)	1.27e-11	0.090(0.074)	0.220	0.003(0.086)	0.973	---	---	0.027(0.056)	0.622	0.034(0.063)	0.589
rs537544	C/T	0.087(0.015)	8.37e-09	-0.017(0.089)	0.852	-0.028(0.103)	0.790	0.026(0.022)	0.245	0.037(0.068)	0.588	-0.144(0.078)	0.065
rs542907	A/G	0.081(0.015)	3.96e-08	-0.073(0.093)	0.434	-0.015(0.108)	0.889	0.081(0.023)	0.000	0.051(0.072)	0.480	-0.115(0.082)	0.159
rs5763842	A/G	0.150(0.015)	1.49e-23	0.045(0.063)	0.477	0.019(0.072)	0.791	0.025(0.013)	0.045	-0.089(0.039)	0.024	0.033(0.045)	0.453
rs6000602	A/C	0.102(0.018)	6.16e-09	0.050(0.095)	0.596	0.215(0.110)	0.051	-0.027(0.021)	0.212	-0.006(0.067)	0.930	-0.061(0.076)	0.420
rs60888743	G/A	0.137(0.017)	9.82e-16	0.008(0.062)	0.900	0.025(0.073)	0.732	---	---	-0.029(0.047)	0.538	-0.010(0.054)	0.858
rs61839660	T/C	0.371(0.031)	3.49e-32	-0.051(0.034)	0.132	-0.033(0.040)	0.407	-0.004(0.008)	0.659	-0.032(0.026)	0.229	0.048(0.029)	0.104
rs705704	A/G	0.196(0.015)	1.89e-37	-0.021(0.041)	0.604	-0.037(0.048)	0.432	---	---	---	---	-0.058(0.035)	0.097
rs7237497	T/C	0.177(0.020)	1.17e-18	-0.071(0.058)	0.221	-0.001(0.067)	0.987	0.014(0.014)	0.314	-0.096(0.044)	0.028	0.047(0.050)	0.341
rs73069541	G/A	0.107(0.018)	3.07e-09	-0.018(0.083)	0.831	-0.110(0.097)	0.258	---	---	-0.024(0.064)	0.702	0.208(0.072)	0.004
rs8011031	G/A	0.090(0.015)	5.95e-09	0.115(0.168)	0.493	0.161(0.205)	0.432	0.001(0.023)	0.964	-0.006(0.072)	0.939	0.005(0.081)	0.952
rs8013873	T/C	0.163(0.023)	4.77e-13	-0.071(0.060)	0.243	-0.051(0.070)	0.467	-0.028(0.015)	0.057	-0.014(0.049)	0.764	-0.049(0.052)	0.350
rs8062123	G/T	0.135(0.021)	6.60e-11	-0.118(0.139)	0.397	-0.004(0.168)	0.980	---	---	-0.096(0.061)	0.116	0.069(0.062)	0.270
rs9517712	T/C	0.096(0.016)	4.26e-09	0.110(0.092)	0.229	0.210(0.107)	0.049	-0.014(0.022)	0.520	0.002(0.069)	0.976	0.010(0.079)	0.898
rs9911533	C/T	0.087(0.015)	5.14e-09	-0.075(0.089)	0.394	0.059(0.103)	0.571	0.019(0.022)	0.384	-0.139(0.068)	0.040	-0.032(0.078)	0.685
rs9976073	C/T	0.094(0.017)	1.13e-08	0.040(0.090)	0.657	-0.019(0.104)	0.856	0.026(0.022)	0.245	-0.018(0.068)	0.791	0.138(0.079)	0.080

rs3184504	T/C	0.218(0.015)	4.62e-49	---	---	---	---	0.035(0.012)	0.004	-0.088(0.027)	0.001	0.027(0.043)	0.523

FN: femoral neck; LS: lumbar spine; TB: total body; SE: standard error.

Table S7. **Results of two-sample Mendelian randomization analyses of type 2 diabetes on bone mineral density/fracture.**

SNP	Effect allele/ Other allele	SNP & Type 2 diabetes		SNP & bone mineral density associations									
				FN-BMD		LS-BMD		Heel-BMD		TB-BMD		Fracture	
		Beta (SE)	*P*	Beta (SE)	*P*	Beta (SE)	*P*	Beta (SE)	*P*	Beta (SE)	*P*	Beta (SE)	*P*
**Type 2 diabetes**													
rs10507349	G/A	0.053(0.009)	1.87e-09	0.064(0.166)	0.701	-0.117 (0.194)	0.547	-0.035 (0.042)	0.403	0.040 (0.126)	0.754	0.164 (-0.147)	0.265
rs10811661	T/C	0.174(0.010)	1.57e-68	0.068(0.058)	0.235	0.059 (0.067)	0.376	-0.023 (0.014)	0.100	---	---	0.044 (0.050)	0.378
rs10842994	C/T	0.069(0.010)	8.87e-12	-0.017 (0.139)	0.904	-0.166 (0.161)	0.303	0.063 (0.034)	0.061	-0.170 (0.107)	0.114	0.198 (0.119)	0.096
rs1111875	C/T	0.102(0.008)	5.59e-39	-0.110 (0.076)	0.149	-0.104 (0.089)	0.240	0.014 (0.018)	0.460	-0.116 (0.057)	0.042	0.045 (0.065)	0.489
rs11123406	T/C	0.042(0.007)	9.10e-09	-0.212 (0.185)	0.251	-0.073 (0.216)	0.735	---	---	-0.095 (0.140)	0.498	0.012 (0.163)	0.943
rs11257655	T/C	0.092(0.008)	3.29e-29	0.089 (0.100)	0.372	0.066 (0.117)	0.571	-0.041 (0.025)	0.094	0.008 (0.075)	0.919	-0.105 (0.087)	0.230
rs11671664	A/G	0.076(0.011)	2.80e-12	0.229 (0.165)	0.166	0.473 (0.190)	0.013	-0.033 (0.041)	0.418	0.376 (0.122)	0.002	-0.224 (0.144)	0.120
rs11708067	A/G	0.095(0.010)	4.68e-20	0.128 (0.096)	0.184	0.057 (0.112)	0.609	---	---	0.033 (0.071)	0.644	-0.092 (0.080)	0.249
rs11787792	A/G	0.108(0.015)	2.37e-12	0.115 (0.076)	0.129	0.100 (0.089)	0.259	0.004 (0.018)	0.817	0.136 (0.058)	0.020	0.021 (0.064)	0.749
rs12571751	A/G	0.067(0.007)	1.66e-20	0.069 (0.119)	0.560	0.092 (0.130)	0.480	0.041 (0.028)	0.136	0.021 (0.085)	0.806	0.151 (0.098)	0.122
rs12681990	C/T	0.052(0.009)	2.28e-09	0.164 (0.198)	0.408	-0.209 (0.229)	0.362	---	---	0.267 (0.146)	0.067	-0.045 (0.172)	0.796
rs12970134	A/G	0.062(0.009)	2.68e-12	0.170 (0.140)	0.223	0.181 (0.163)	0.267	---	---	0.265 (0.105)	0.012	0.039 (0.119)	0.741
rs13266634	C/T	0.109(0.008)	4.29e-42	0.022 (0.072)	0.761	-0.040 (0.084)	0.631	0.032 (0.018)	0.081	0.012 (0.058)	0.837	-0.043 (0.065)	0.510
rs13292136	C/T	0.094(0.016)	1.95e-09	-0.112 (0.160)	0.483	0.029 (0.186)	0.875	0.118 (0.039)	0.003	0.116 (0.117)	0.322	0.181 (0.139)	0.193
rs1359790	G/A	0.071(0.009)	1.35e-15	0.169 (0.120)	0.160	-0.137 (0.140)	0.328	---	---	0.154 (0.090)	0.089	0.060 (0.102)	0.555
rs1470579	C/A	0.130(0.009)	1.44e-45	0.094 (0.064)	0.146	0.044 (0.125)	0.726	---	---	0.098 (0.047)	0.036	-0.065 (0.054)	0.231
rs1552224	A/C	0.097(0.011)	2.55e-17	0.033 (0.108)	0.757	-0.190 (0.187)	0.308	0.085 (0.026)	0.001	0.108 (0.081)	0.184	0.103 (0.093)	0.266
rs17106184	G/A	0.080(0.014)	1.20e-08	-0.388 (0.161)	0.016	-0.005 (0.183)	0.979	---	---	0.266 (0.123)	0.030	-0.183 (0.141)	0.193
rs17168486	T/C	0.065(0.009)	1.09e-12	0.126 (0.158)	0.425	-0.127 (0.187)	0.497	-0.013 (0.037)	0.721	0.122 (0.114)	0.286	0.146 (0.133)	0.273
rs2191349	T/G	0.048(0.008)	1.35e-09	-0.188 (0.161)	0.242	-0.165 (0.085)	0.053	---	---	-0.029 (0.119)	0.806	0.275 (0.137)	0.044

rs2237892	C/T	0.215(0.013)	2.42e-63	-0.116 (0.073)	0.112	0.547 (0.208)	0.008	-0.012 (0.017)	0.474	0.088 (0.054)	0.106	-0.083 (0.062)	0.181
rs2296172	G/A	0.050(0.010)	6.07e-07	-0.091 (0.178)	0.610	0.120 (0.141)	0.397	---	---	0.306 (0.140)	0.029	-0.382(0.160)	0.017
rs231362	G/A	0.064(0.010)	1.83e-11	0.046 (0.122)	0.705	0.453 (0.186)	0.015	-0.016 (0.029)	0.573	0.161 (0.092)	0.081	-0.020 (0.102)	0.843
rs2421016	C/T	0.048(0.007)	3.86e-11	---	---	---	---	0.085 (0.038)	0.026	---	---	-0.591 (0.136)	0.000
rs2796441	G/A	0.057(0.007)	1.58e-14	-0.146 (0.136)	0.283	-0.052 (0.158)	0.743	-0.045 (0.033)	0.175	-0.074 (0.105)	0.484	-0.019 (0.116)	0.871
rs2867125	C/T	0.061(0.010)	1.73e-09	0.032 (0.165)	0.848	0.051 (0.193)	0.791	---	---	0.346 (0.126)	0.006	-0.118 (0.142)	0.407
rs2925979	T/C	0.047(0.009)	3.75e-08	0.431 (0.174)	0.013	0.321 (0.202)	0.111	-0.093 (0.043)	0.030	0.194 (0.134)	0.149	-0.365 (0.152)	0.016
rs3130501	G/A	0.063(0.009)	4.09e-12	0.046 (0.155)	0.764	0.089 (0.175)	0.609	-0.013 (0.033)	0.697	0.327 (0.114)	0.004	0.018 (0.119)	0.882
rs329122	A/G	0.043(0.007)	2.90e-09	-0.059 (0.194)	0.759	-0.203 (0.210)	0.333	-0.066 (0.044)	0.127	-0.063 (0.133)	0.636	-0.046 (0.154)	0.764
rs340874	C/T	0.049(0.008)	6.02e-10	0.196 (0.154)	0.204	0.003 (0.179)	0.989	0.085 (0.038)	0.025	0.057 (0.116)	0.623	0.155 (0.135)	0.250
rs3794991	T/C	0.077(0.013)	4.08e-09	-0.187 (0.322)	0.562	-0.460 (0.395)	0.244	0.106 (0.043)	0.014	0.264 (0.140)	0.060	0.155 (0.153)	0.310
rs3923113	A/C	0.071(0.010)	1.26e-13	0.192 (0.112)	0.085	0.178 (0.130)	0.172	-0.028 (0.027)	0.300	0.058 (0.082)	0.480	-0.030 (0.096)	0.757
rs4420638	A/G	0.077(0.014)	8.82e-08	-0.070 (0.140)	0.618	-0.288 (0.163)	0.077	-0.022 (0.031)	0.481	-0.219 (0.105)	0.037	0.084 (0.108)	0.438
rs4430796	G/A	0.083(0.011)	2.95e-15	0.107 (0.098)	0.278	0.070 (0.109)	0.521	0.043 (0.022)	0.053	0.170 (0.071)	0.017	0.058 (0.079)	0.465
rs4457053	G/A	0.066(0.011)	4.82e-10	0.146 (0.123)	0.235	0.071 (0.144)	0.620	0.060 (0.030)	0.047	0.085 (0.094)	0.366	-0.151 (0.106)	0.154
rs459193	G/A	0.075(0.008)	5.08e-19	0.152 (0.114)	0.183	-0.034 (0.133)	0.801	---	---	0.077 (0.087)	0.372	-0.291 (0.100)	0.004
rs4689388	A/G	0.075(0.009)	1.31e-15	0.013 (0.105)	0.903	-0.053 (0.122)	0.666	-0.014 (0.025)	0.584	0.056 (0.077)	0.469	0.033 (0.088)	0.709
rs4812829	A/G	0.057(0.009)	3.75e-10	0.071 (0.177)	0.687	0.237 (0.204)	0.246	0.111 (0.045)	0.013	0.163 (0.133)	0.221	-0.107 (0.157)	0.497
rs516946	C/T	0.086(0.009)	2.04e-21	0.020 (0.101)	0.847	0.037 (0.117)	0.753	0.006 (0.025)	0.814	0.047 (0.078)	0.550	0.021 (0.089)	0.818
rs5215	C/T	0.068(0.007)	1.36e-20	0.023 (0.111)	0.836	-0.073 (0.129)	0.572	0.021 (0.028)	0.460	0.051 (0.087)	0.553	-0.097 (0.100)	0.336
rs576674	G/A	0.072(0.010)	1.07e-12	0.049 (0.143)	0.731	0.077 (0.167)	0.644	-0.016 (0.034)	0.645	-0.133 (0.104)	0.201	0.067 (0.122)	0.580
rs622217	T/C	0.052(0.009)	5.81e-09	0.041 (0.149)	0.782	-0.041 (0.174)	0.816	-0.018 (0.035)	0.601	-0.094 (0.112)	0.398	-0.014 (0.126)	0.911
rs6813195	C/T	0.061(0.008)	1.61e-15	0.286 (0.135)	0.035	0.389 (0.158)	0.014	---	---	0.198 (0.103)	0.055	-0.098 (0.123)	0.423
rs702634	A/G	0.050(0.008)	3.09e-09	0.263 (0.162)	0.104	0.459 (0.188)	0.015	0.113 (0.040)	0.005	-0.050 (0.124)	0.687	-0.043 (0.142)	0.760
rs7111341	T/C	0.069(0.010)	2.13e-11	-0.050 (0.130)	0.698	0.068 (0.145)	0.640	0.065 (0.030)	0.029	-0.133 (0.096)	0.163	-0.009 (0.107)	0.931
rs7172432	A/G	0.057(0.010)	4.78e-09	-0.157 (0.134)	0.239	-0.078 (0.155)	0.615	-0.061 (0.033)	0.065	-0.102 (0.100)	0.309	0.092 (0.116)	0.427

rs7178572	G/A	0.065(0.008)	9.58e-18	-0.063 (0.127)	0.619	-0.107 (0.148)	0.468	-0.059 (0.032)	0.060	0.023 (0.095)	0.809	-0.323 (0.111)	0.004
rs7578326	A/G	0.072(0.010)	2.25e-13	-0.051 (0.112)	0.650	0.237 (0.130)	0.068	0.058 (0.027)	0.030	-0.004 (0.082)	0.959	0.172 (0.096)	0.073
rs7578597	T/C	0.099(0.015)	5.85e-11	---	---	---	---	---	---	---	---	0.173 (0.106)	0.104
rs7612463	C/A	0.098(0.013)	4.27e-14	-0.086 (0.120)	0.473	-0.245 (0.138)	0.074	-0.021 (0.030)	0.494	-0.009 (0.088)	0.917	-0.106(0.107)	0.319
rs780094	C/T	0.064(0.007)	2.07e-17	0.235 (0.120)	0.050	0.103 (0.139)	0.457	0.068 (0.029)	0.021	---	---	-0.025 (0.105)	0.811
rs7903146	T/C	0.301(0.010)	1.33e-219	0.042 (0.028)	0.141	0.345 (0.140)	0.013	0.037 (0.007)	0.000	0.055 (0.021)	0.008	0.036 (0.024)	0.128
rs8042680	A/C	0.057(0.009)	1.58e-09	-0.041 (0.154)	0.792	0.064 (0.184)	0.729	0.060 (0.036)	0.093	0.039 (0.105)	0.714	0.099 (0.125)	0.426
rs8050136	A/C	0.106(0.008)	2.78e-39	-0.121 (0.075)	0.105	0.037 (0.084)	0.655	---	---	-0.128 (0.055)	0.019	-0.147(0.063)	0.020
rs864745	T/C	0.070(0.008)	3.93e-19	0.092 (0.107)	0.388	0.239 (0.125)	0.055	---	---	---	---	0.049 (0.093)	0.603
rs9505118	A/G	0.044(0.008)	1.86e-08	-0.208 (0.172)	0.226	0.033 (0.201)	0.870	---	---	0.082 (0.130)	0.528	0.049 (0.151)	0.744
rs9940149	G/A	0.052(0.009)	1.70e-09	-0.256 (0.186)	0.169	-0.166 (0.216)	0.442	-0.049 (0.047)	0.296	-0.065 (0.142)	0.646	0.124 (0.164)	0.449

FN: femoral neck; LS: lumbar spine; TB: total body; SE: standard error.

Table S8. Results of two-sample Mendelian randomization analyses of HbA1c on bone mineral density/fracture.

SNP	Effect allele/ Other allele	SNP & HbA1c		SNP & bone mineral density associations									
				FN-BMD		LS-BMD		Heel-BMD		TB-BMD		Fracture	
		Beta (SE)	*P*	Beta (SE)	*P*	Beta (SE)	*P*	Beta (SE)	*P*	Beta (SE)	*P*	Beta (SE)	*P*
**HbA1c**													
rs1046896	T/C	0.035(0.003)	1.57e-26	0.419 (0.235)	0.074	0.539 (0.273)	0.048	-0.067 (0.057)	0.242	0.054 (0.174)	0.755	0.019 (0.202)	0.923
rs1387153	T/C	0.028(0.004)	3.96e-11	0.293 (0.305)	0.338	0.330 (0.355)	0.352	0.336 (0.072)	3.304	0.268 (0.229)	0.241	-0.116 (0.257)	0.653
rs16926246	C/T	0.089(0.004)	3.11e-54	-0.107 (0.129)	0.409	0.046 (0.151)	0.759	-0.088 (0.031)	0.004	-0.071 (0.099)	0.474	-0.063 (0.109)	0.562
rs1799884	T/C	0.038(0.004)	1.45e-20	-0.072 (0.266)	0.787	-0.149 (0.298)	0.616	-0.025 (0.063)	0.695	0.026 (0.195)	0.893	0.090 (0.225)	0.690
rs1800562	G/A	0.063(0.007)	2.59e-20	0.040 (0.233)	0.862	-0.053 (0.272)	0.846	0.184 (0.054)	0.001	0.071 (0.189)	0.705	0.033 (0.193)	0.865
rs2779116	T/C	0.024(0.004)	2.75e-09	-0.132 (0.362)	0.716	0.387 (0.358)	0.358	-0.244 (0.086)	0.005	0.096 (0.271)	0.723	-0.487 (0.308)	0.114
rs4737009	A/G	0.027(0.004)	6.11e-12	0.155 (0.327)	0.636	-0.027 (0.379)	0.943	-0.112 (0.081)	0.165	0.293 (0.252)	0.245	0.119 (0.285)	0.676
rs552976	G/A	0.047(0.003)	8.16e-18	0.327 (0.171)	0.458	0.038 (0.200)	0.850	0.058 (0.041)	0.157	0.151 (0.126)	0.229	0.069 (0.145)	0.634
rs6474359	T/C	0.058(0.011)	1.18e-08	-0.562 (0.353)	0.112	0.079 (0.412)	0.847	-0.072 (0.084)	0.391	-0.007 (0.252)	0.978	-0.209 (0.296)	0.480
rs7998202	G/A	0.031(0.005)	5.24e-09	-0.270 (0.353)	0.444	-0.414 (0.411)	0.313	0.109 (0.086)	0.204	-0.174 (0.271)	0.520	0.321 (0.302)	0.287

FN: femoral neck; LS: lumbar spine; TB: total body; SE: standard error.
